# Antioxidant Nanoparticles for Concerted Inhibition of α-Synuclein Fibrillization, and Attenuation of Microglial Intracellular Aggregation and Activation

**DOI:** 10.3389/fbioe.2020.00112

**Published:** 2020-02-21

**Authors:** Nanxia Zhao, Xue Yang, Hannah R. Calvelli, Yue Cao, Nicola L. Francis, Rebecca A. Chmielowski, Laurie B. Joseph, Zhiping P. Pang, Kathryn E. Uhrich, Jean Baum, Prabhas V. Moghe

**Affiliations:** ^1^Department of Chemical and Biochemical Engineering, Rutgers, The State University of New Jersey, Piscataway, NJ, United States; ^2^Department of Chemistry and Chemical Biology, Rutgers, The State University of New Jersey, Piscataway, NJ, United States; ^3^Department of Molecular Biology and Biochemistry, Rutgers, The State University of New Jersey, Piscataway, NJ, United States; ^4^Department of Chemistry, University of California, Riverside, Riverside, CA, United States; ^5^Department of Biomedical Engineering, Rutgers, The State University of New Jersey, Piscataway, NJ, United States; ^6^Department of Pharmacology and Toxicology, Rutgers, The State University of New Jersey, Piscataway, NJ, United States; ^7^Department of Neuroscience and Cell Biology, Child Health Institute of New Jersey, Rutgers Robert Wood Johnson Medical School, New Brunswick, NJ, United States

**Keywords:** Parkinson’s disease, microglia, alpha synuclein, ferulic acid, tannic acid, nanomedicine

## Abstract

Parkinson’s Disease is characterized by the loss of dopaminergic neurons in the *substantia nigra pars compacta*, the extracellular accumulation of toxic α-synuclein (αSYN) aggregates, and neuroinflammation. Microglia, resident macrophages of the brain, are one of the critical cell types involved in neuroinflammation. Upon sensing extracellular stimuli or experiencing oxidative stress, microglia become activated, which further exacerbates neuroinflammation. In addition, as the first line of defense in the central nervous system, microglia play a critical role in αSYN clearance and degradation. While the role of microglia in neurodegenerative pathologies is widely recognized, few therapeutic approaches have been designed to target both microglial activation and αSYN aggregation. Here, we designed nanoparticles (NPs) to deliver aggregation-inhibiting antioxidants to ameliorate αSYN aggregation and attenuate activation of a pro-inflammatory microglial phenotype. Ferulic acid diacid with an adipic acid linker (FAA) and tannic acid (TA) were used as shell and core molecules to form NPs via flash nanoprecipitation. These NPs showed a strong inhibitory effect on αSYN fibrillization, significantly diminishing αSYN fibrillization *in vitro* compared to untreated αSYN using a Thioflavin T assay. Treating microglia with NPs decreased overall αSYN internalization and intracellular αSYN oligomer formation. NP treatment additionally lowered the *in vitro* secretion of pro-inflammatory cytokines TNF-α and IL-6, and also attenuated nitric oxide and reactive oxygen species production induced by αSYN. NP treatment also significantly decreased Iba-1 expression in αSYN-challenged microglia and suppressed nuclear translocation of nuclear factor kappa B (NF-κB). Overall, this work lays the foundation for an antioxidant-based nanotherapeutic candidate to target pathological protein aggregation and neuroinflammation in neurodegenerative diseases.

## Introduction

Parkinson’s Disease (PD) is one of the most common neurodegenerative disorders and affects 6 million individuals globally (de Rijk et al., 1997; Gbd 2016 Parkinson’s Disease Collaborators, 2018). The national economic burden of PD was approximately $14.4 billion in 2010 in the United States. and is projected to increase as the population ages (Kowal et al., 2013; Kalia and Lang, 2015; Gbd 2016 Parkinson’s Disease Collaborators, 2018). The progressive loss of dopaminergic (DA) neurons in the substantia nigra pars compacta (SNc) and the abnormal accumulation and pathological aggregation of extracellular α-synuclein (αSYN) are hallmarks of PD. The αSYN protein was first found in Lewy bodies, abnormal aggregates of αSYN that develop within neurons in PD patients (Lucking and Brice, 2000). The aggregation of αSYN originates from soluble monomers, dimers, and oligomers, which develop into protofibrils and insoluble fibrils composed of β-sheets and amyloid-like filaments (Lashuel et al., 2013). Moreover, another key feature of PD pathology is neuroinflammation, in which microglial activation is one of the key phenomena (Graeber et al., 2011). Microglia, as the resident immune cells in the brain, are the first line of defense in the central nervous system (CNS) and can generate adaptive or innate immune responses upon detection of invading pathogens (Olson and Miller, 2004).

αSYN has been shown to play a role in regulating synaptic transmission between neurons and synaptic plasticity (Lashuel et al., 2013). Various conformational forms of αSYN including oligomeric and fibril states exist in dynamic equilibrium (Ghiglieri et al., 2018). However, this equilibrium can be disrupted and shifted toward aggregated forms due to oxidative stress, mutant forms of αSYN such as A53T which have been linked to familial PD, post-translational protein modifications, and high local concentrations of αSYN (Stefanis, 2012; Pineda and Burre, 2017). Under normal conditions, microglia scavenge and regulate responses to cell debris and foreign biomolecules in the brain and participate in the clearance of pathological protein aggregates (Su et al., 2008). However, high extracellular concentrations of αSYN promote the development of a pro-inflammatory microglial phenotype, which impairs the normal αSYN clearance pathway, leading to the intracellular aggregation of toxic αSYN oligomer species (Bengoa-Vergniory et al., 2017). These αSYN oligomer species can then be transmitted to other microglia and neurons, as well as other cells in the CNS, leading to widespread neurotoxicity and propagation of further toxic αSYN aggregation (Longhena et al., 2017; Fields et al., 2019).

Microglial activation is one of the main neuroinflammatory mechanisms that contributes to neuronal degeneration, as shown by post-mortem and *in vivo* studies in PD (Hirsch and Hunot, 2009; Moore et al., 2018). Release of pro-inflammatory cytokines and reactive free radicals such as tumor necrosis factor (TNF-α), interleukin-6 (IL-6) nitric oxide (NO), reactive oxygen species (ROS) and extracellular vesicles containing toxic protein aggregates resulting from microglial activation can lead to the degeneration and death of neighboring healthy neurons, particularly DA neurons (Hoenen et al., 2016; Subramaniam and Federoff, 2017). The death of DA neurons leads to accumulation of αSYN in the extracellular space, resulting in a destructive cycle of microglial activation and neurodegeneration (Marin-Teva et al., 2011).

Polyphenol antioxidants such as tannic acid (TA) have shown the ability to inhibit αSYN oligomerization and fibrillization (Ono and Yamada, 2006; Caruana et al., 2011; Takahashi et al., 2015; Javed et al., 2018). Moreover, polyphenols like ferulic acid are effective scavengers for free radicals and superoxide anions and are used to prevent lipid peroxidation as food additives (Srinivasan et al., 2007). Although there is an ongoing search for improved PD therapeutics which can halt or reverse the disease progression rather than treat the symptoms, the complicated mechanism of PD challenges the development of effective therapeutics focused on unitary targets (Stoker et al., 2018). A multi-target therapeutic approach that can modulate more than one pathological feature may be more promising than single-target therapeutics to slow or halt disease progression.

We propose here a functionally combinatorial approach using a nanoparticle (NP) formulation containing two polyphenol antioxidants to target (1) toxic protein aggregation, a key biomarker of PD and (2) its subsequent downstream effect: neuroinflammation. Specifically, we hypothesized that tannic acid, which has been shown to be a strong αSYN aggregation inhibitor, and ferulic acid diacid molecule with adipic linker, a potent anti-inflammatory agent, can be combined into a multifunctional, nanoparticle formulation to attenuate microglial activation while enabling normal αSYN clearance and inhibiting intracellular αSYN oligomerization. Compared with traditional antioxidant molecules, NP formulations offer a more stable structure and sustained release of active compounds, as well as the potential for targeting *in vivo*. We demonstrate the ability of antioxidant NPs to inhibit αSYN fibrillization and to decrease intracellular αSYN oligomerization in microglia challenged with high concentration of extracellular αSYN, ameliorating microglial oxidative stress.

## Materials and Methods

### Antioxidant Molecules

**FIGURE 1:**
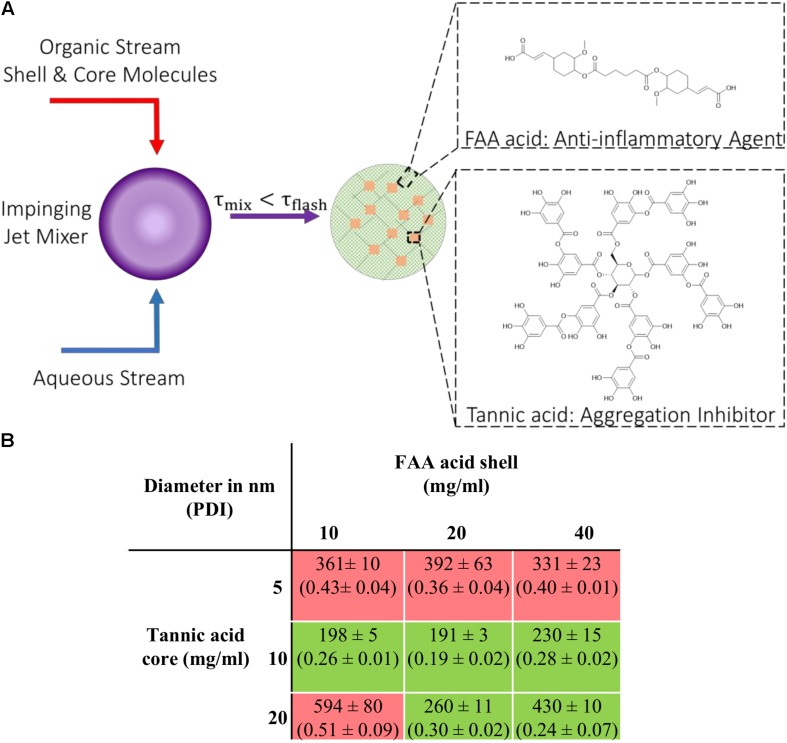
Nanoparticle (NPs) fabrication via flash nanoprecipitation (FNP) **(A)** Schematic description of the FNP process with components required to form stable colloidal NPs. **(B)** Table listing NPs fabricated with different shell-to-core concentration ratios investigated; hydrodynamic diameters (nm) and polydispersity index (PDI). The green box indicates successful NP formation (PDI < 0.3) and the red box indicates failed attempts (PDI > 0.3). Data represent mean ± SEM; *n* = 3.

FA-adipic diacid (FAA acid) was synthesized and characterized as previously reported in the literature (Ouimet et al., 2013, 2015). Briefly, t-Butyl FA was synthesized by adding tertiary butanol to Meldrum’s acid dissolved in toluene, after which vanillin, pyridine and piperidine was added (Ouimet et al., 2013, 2015). The reaction mixture was then diluted in diethyl ether and washed with proper solvents before the organic layer was dried over MgSO_4_ (Ouimet et al., 2013, 2015). t-Butyl FA was then dissolved in dimethylformamide (DMF) where sodium hydride, adipoyl chloride was added before extracting and drying the product (Ouimet et al., 2013). The dried product was then dissolved in dichloromethane (DCM) with added trifluoroacetic acid (TFA) (Ouimet et al., 2013). The solution was left to stir overnight and the final product of FAA acid was collected after isolation via vacuum filtration and dried in vacuo for 24 h (Ouimet et al., 2013). The chemical structure of FAA acid is shown in Figure 1. Tannic acid was purchased from Sigma Aldrich.

### NP Fabrication

NPs were fabricated using a flash nanoprecipitation technique as previously described (York et al., 2012; Lewis et al., 2015). Briefly, shell and core molecules were dissolved in DMSO at varying concentrations and were mixed at 50/50 % (v/v) ratio in a final volume of 500 μL. A confined impinging jet mixer was used to rapidly mix an aqueous water stream and organic phase stream containing shell and core molecules (Markwalter et al., 2019). The exit stream was collected in 9× volume of water and the nanoparticles were dialyzed against water using a 3.5 kDa MW cutoff dialysis cassette. NPs were characterized with DLS using a Malvern-Zetasizer Nano ZS90 series DLS detector with a max 4 mW He-Ne laser operating at 633 nm using general purpose resolution mode as previously described (York et al., 2012).

### Cell Culture

BV2 microglia, kindly provided by Drs. Bin Liu (University of Florida) and Jason Richardson (Northeast Ohio Medical University), were cultured in DMEM (Gibco) supplemented with 10% fetal bovine serum (Atlanta Biologics) and 1% Penicillin-Streptomycin (Thermo Fisher Scientific).

### Kinetics of αSYN Fibrillization

A fibrillization assay was performed using 100 μL of 1 mg/mL monomeric αSYN (rPeptide). Samples containing either αSYN alone or αSYN + NPs at 1:10 volume ratio were loaded with 20 μM Thioflavin T (ThT) (Acros Organics) into 96 well clear bottomed plates (Corning), sealed with Axygen sealing tape (Corning), and shaken at 600 rpm at 37°C for at least 63 h. Samples were taken at various time points and a POLARstar Omega plate reader (BMG Labtech) was used to monitor the increase in ThT intensity, with fluorescence intensity measured at excitation −450 nm and emission −485 nm. This protocol was adapted from the literature (Moriarty et al., 2017).

### MTT Cell Proliferation Assay

A Vybrant MTT assay was performed following the manufacturer’s protocol (Thermo Fisher Scientific). The NPs’ effect on BV2 cell viability was evaluated at 48 h and 72 h. Briefly, BV2 microglial cells were treated with NPs for two consecutive 24 h treatments (a total of 48 h) or three consecutive 24 h treatments (a total of 72 h) with one wash after each 24 h treatment, after which treatment media was removed and replaced with 100 μL fresh medium containing NPs. MTT stock solution was added to each well and incubated at 37°C for 4 h. Then, 100 μL of SDS-HCl solution was added to each well and mixed via pipetting. The samples were then incubated for an additional 4 h at 37°C before absorbance of samples were read at 570 nm using a Tecan Infinite M200 Pro microplate reader. The absorbance of MTT dye alone was subtracted as background from all readings and the sample absorbance reading was normalized to an untreated control.

### Nanoparticle Uptake Pathway

To investigate the potential pathways of NP internalization in microglia, BV2 microglial cells were plated at 20,000 cells per well in a 96 well plate and were pretreated with different endocytosis inhibitors for 1 h. Chlorpromazine (5 μM, Millipore Sigma) was used to block clathrin-mediated endocytosis, 50 μM amiloride hydrochloride hydrate (Millipore Sigma) was used as a macropinocytosis inhibitor, 200 μM genistein (Millipore Sigma) as a caveolae-mediated endocytosis inhibitor, and 5 mM methyl-β-cyclodextrin was used to block lipid raft-mediated endocytosis. These concentrations were chosen based on relevant literature (Li and Monteiro-Riviere, 2016; Abou Matar and Karam, 2018; Dalal and Jana, 2018). Next, pretreatment was removed and [FAA:TA] NPs fluorescently labeled with 1,1′-Dioctadecyl-3,3,3′,3′-Tetramethylindocarbocyanine Perchlorate (Thermo Fisher Scientific) were added with corresponding inhibitors in media. After 3 h incubation, cells were washed with phosphate-buffered saline (PBS) to remove extracellular NPs and fixed with 4% paraformaldehyde (PFA) followed by two PBS washes. Cells were imaged on a Zeiss LSM 780 confocal microscope using a 20× objective. Intracellular fluorescence quantification was performed using NIH-ImageJ software and normalized to the cell count within the field of view.

### αSYN Internalization

BV2 microglial cells were plated at 20,000 per well in a 96 well plate and allowed to adhere overnight. Cells were then pre-incubated with NPs or control compounds for 24 h, followed by 24 h co-incubation with 5 μM monomeric αSYN (rPeptide) or with 20 wt% HiLyte Fluor 488 labeled monomeric αSYN (Anaspec) for imaging purposes. For labeled αSYN studies, cells were fixed with 4% PFA followed by two washes with PBS to remove residual PFA as well as extracellular αSYN. Cells were imaged on a Zeiss LSM 780 confocal microscope using a 20× objective. Intracellular fluorescence quantification was performed using NIH-ImageJ software and normalized to the cell count within the field of view.

### Cytokine and Nitric Oxide Assays

BV2 microglia were plated at 50,000 cells per well in a 96 well plate and allowed to adhere overnight. Cells were treated with 5 μM A53T αSYN with or without the addition of NPs or compound controls. Supernatants from the cultured microglia were collected after 24 h and assayed for TNF-α and IL-6 production using ELISA according to the manufacturer’s protocol (R&D systems). Nitric oxide production was determined via quantification of nitrite concentration using Griess reagent (Promega).

### ROS Detection

BV2 microglia were plated at 20,000 per well in a 96 well plate and allowed to adhere overnight. Cells were treated with 10 μM A53T αSYN with or without the addition of NPs or compound controls. After 24 h, intracellular ROS production was quantified with CellROX Deep Red reagent according to the manufacturer’s protocol (Thermo Fisher Scientific). Briefly, cells were stained with 5 μM CellROX reagent for 30 min and then fixed with 4% PFA and washed 3 times with PBS. Cells were then imaged on a Zeiss LSM 780 confocal microscope using a 10× objective. Intracellular CellROX fluorescence quantification was performed using NIH-ImageJ software and normalized to the cell count within the field of view.

### Immunocytochemistry

BV2 microglial cells were treated with 10 μM A53T αSYN and fixed with 4% PFA for 15 min at room temperature and then were simultaneously blocked and permeabilized in blocking buffer containing 1% BSA, 5% normal goat serum (MP Biomedicals) and 0.1% Triton X-100 for 1 h at room temperature. Cells were then incubated with primary antibodies in blocking buffer at 4°C overnight, followed by three 15-min washes with PBS. Cells were incubated with fluorophore-conjugated secondary antibodies (Alexa Fluor 594, Thermo Fisher Scientific) in blocking buffer for 1 h at room temperature, followed by three 15 min washes with PBS. Cells were counterstained with Hoechst 33342 to visualize cell nuclei. Primary antibodies used in this study were anti-CD36 antibody [JC63.1] (Abcam), anti-Iba1 antibody (Wako) and anti-NF-κB p65 (F-6) antibody (Santa Cruz Biotechnology). Fluorescent images of cells were taken with a Zeiss LSM 780 confocal microscope using a 20×, a 40× water-immersion or a 63× oil-immersion objective. Iba1 fluorescence quantification was performed using NIH-ImageJ software and normalized to the cell count within the field of view. A NF-κB nuclear translocation study was conducted as described in previous publications with slight modifications (Trask, 2004; Bagaev et al., 2019). Briefly, a set of ROIs were defined using Hoechst nuclear stain fluorescence and subsequently overlaid on NF-κB fluorescence images after applying same fluorescence threshold to all images. The percentage of nuclear translocation was calculated by taking the ratio of NF-κB fluorescence within the defined ROIs for each individual cell and overall NF-κB fluorescence in the image containing both nuclear and cytoplasm signals.

### Western Blotting

Cell lysates containing intracellular αSYN were collected in PBS, 1% Triton X-100 and 1× phosphatase inhibitor cocktails (Pierce). Samples were mixed with sample buffer containing 62.5 mM Tris, pH 6.8, 10% glycerol, 2% SDS, 2.5% β-mercaptoethanol and 0.025% bromophenol blue, then running on a Bolt 4–12% Bis-Tris Plus gel (Thermo Fisher Scientific) for 90 min at 100 V. Samples were then transferred onto methanol-activated polyvinyl difluoride (PVDF) membrane (Thermo Fisher Scientific) at 300 mA for 90 min at 4°C. PVDF membranes were then blocked in 5% milk (Santa Cruz Biotechnology) and 0.05% Tween 20 (Sigma) in PBS for 1 h with gentle shaking. PVDF membranes were then incubated with primary antibodies in blocking buffer at 4°C overnight and washed five times for 5 min in PBS with 0.05% Tween 20, followed by incubation with secondary antibodies in blocking buffer for 1 h at room temperature. The PVDF membranes were then washed five times for 5 min in PBS with 0.05% Tween 20 and developed using ECL blotting solution (Pierce) for 2 min. PVDF membranes were then developed using Kodak Biomax Carestream light film (Sigma).

### Statistical Analysis

Data are presented as mean ± SEM unless otherwise indicated, from at least 3 independent experiments (*n* ≥ 3). Analysis was performed using one-way ANOVA with Tukey’s *post hoc* test with *p* < 0.05 considered to be statistically significant (GraphPad Prism 8 software). The exact *p* values listed within figure captions are multiplicity adjusted *p* values as computed during Tukey’s *post hoc* test.

## Results

### Dual Component Antioxidant NPs Prevent α-Synuclein Fibrillization

NPs composed of ferulic acid diacid (FAA acid) molecule in the shell and TA in the core were fabricated via flash nanoprecipitation (FNP), where an organic solution containing the hydrophobic compound tannic acid and the more hydrophilic FAA molecule, and an anti-solvent aqueous solution meet at the junction of a confined impinging jet mixer (Figure 1A). The fast mixing speed enables supersaturation of the shell and core materials at the junction and enables the formation of nano-assemblies. Different shell-to-core weight ratios were used during the fabrication process to determine the optimal ratio(s) at which stable and non-aggregating nanoparticles could be made. The range of shell and core material concentrations was chosen by taking two aspects into consideration: stable particle formation and particle size. The total concentration of shell and core has to be above a certain threshold to enable stable particle formation with a polydispersity index (PDI) below 0.3, indicating a relatively monodisperse distribution of individual nanoparticle mass. Higher concentrations of shell and core materials included in the fabrication process lead to larger particle sizes due to steric hindrance. Our results show that stable [FAA:TA] NPs were formed at shell-to-core concentration ratios of 10–10, 20–10, 40–10, 20–20, and 40–20 mg/ml (Figure 1B). All stable NPs that were fabricated had a PDI less than 0.3, with mean diameter ranging from 190–450 nm (Figure 1B). As hypothesized, NP size increased with higher FAA and TA concentrations and the total polymer concentration had to be above a certain threshold to form stable NP structures. NPs formed with 40 mg/ml FAA acid in the shell and 20 mg/ml tannic acid in the core were used for further experiments since these NPs contained the highest concentrations of bioactive antioxidants and would thus be expected to have the strongest bioactivity.

Next, we explored the effect of [FAA:TA] NPs on αSYN fibrillization, conducting a Thioflavin T (ThT) kinetic fibrillization assay using monomeric N-terminal acetylated αSYN (Ac-αSYN). Ac-αSYN was used because acetylation is one of the most common post-translational modifications of αSYN and may have an important functional or pathological role (Iyer et al., 2016). ThT fluorescence is positively correlated with amyloid fibril formation since the binding to fibrillar protein structures stops bond rotation of the ThT molecule in aqueous solutions, and thus recovers its emission at 485 nm once excited (Naiki et al., 1989).

**FIGURE 2:**
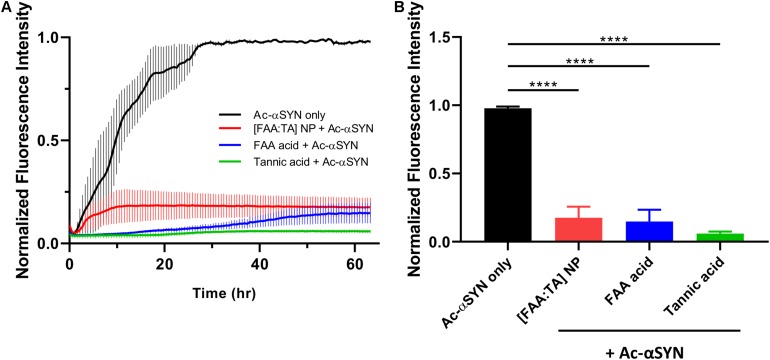
Effect of NPs on acetylated αSYN fibrillization kinetics **(A)** Thioflavin T kinetic assay conducted with acetylated αSYN monomer over 63 h. **(B)** End point ThT fluorescence at 63 h. [FAA:TA] NPs show a strong fibrillization inhibition effect. Data are presented as mean ± SEM; *n* = 3; ****P < 0.0001 for all pairwise comparison shown on graph by one-way ANOVA.

The fluorescence signal of ThT demonstrated a characteristic sigmoidal curve and has three parts: a lag phase, a growth phase and an equilibrium phase. [FAA:TA] NPs significantly shortened the Ac-αSYN growth phase, indicating its efficacy in controlling Ac-αSYN fibrillization. The addition of free FAA acid and tannic acid controls also significantly inhibited Ac-αSYN fibrillization proceeding to equilibrium. Based on the results of the Thioflavin T aggregation assay, the mechanism of inhibition of NPs on preventing aggregation is likely due to the release of active components FAA acid and TA as the ThT curve shows a concave shape – NP’s inhibition action strengthens as more FAA acid and TA is released (the rate at which fibril formation is slowing down). The end point ThT fluorescence at 63 h in equilibrium phase shows an increase of only 17.6% for Ac-αSYN + [FAA:TA] NPs and only 5.9% for Ac-αSYN + tannic acid, while there was no significant difference when comparing the addition of NPs, FAA acid, or tannic acid (Figure 2B). The end point fluorescence is 83% lower with the introduction of [FAA:TA] NPs into Ac-αSYN compared with Ac-αSYN control, indicating that [FAA:TA] NPs can significantly reduce Ac-αSYN fibrillization and slow down the aggregation kinetics (Figure 2B). Similar fibrillization kinetics were also observed using wild type αSYN (Supplementary Figure S1). We also examined the effects of ferulic acid, which is the breakdown product of FAA acid. Ferulic acid lowered the end point ThT fluorescence to 78.7% of Ac-αSYN alone, however, there was a significant difference in end point fluorescence compared to the addition of NPs or tannic acid (Supplementary Figure S2).

### Antioxidant NPs Attenuate Intracellular α-Synuclein Oligomerization in Microglia

**FIGURE 3:**
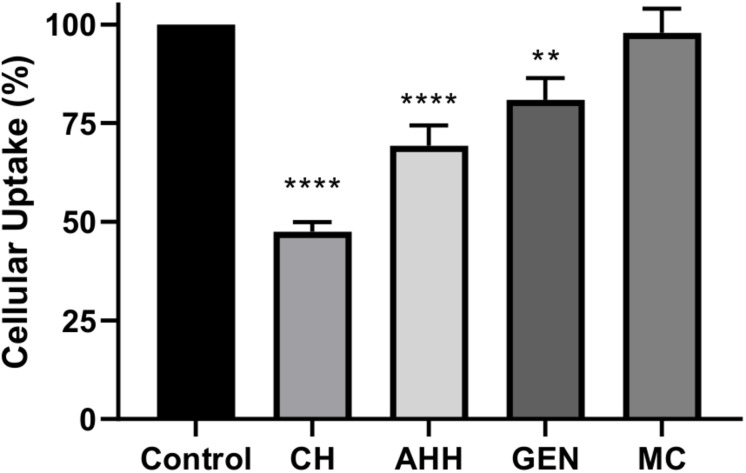
[FAA:TA] NP uptake pathway studies with different endocytosis inhibitors. BV2 microglia cells were pretreated with different endocytosis pathway inhibitors at corresponding concentration for 1 h, after which the treatment were removed and fluorescently labeled NPs was added in the presence of pathway inhibitors for 3 h. CH: Chlorpromazine hydrochloride; AHH: Amiloride hydrochloride hydrate; GEN: Genistein; MC: Methyl-β-cyclodextrin (Error bar shows standard error of *n* = 3). ****P < 0.0001 for control vs. CH and control vs. AHH, **P = 0.0049 for control vs. GEN by one-way ANOVA.

We next investigated whether the effect of NPs on inhibiting αSYN aggregation applies within microglial cells and whether NPs can modulate αSYN content in microglia cultures. The immortalized BV2 microglial cell line has been widely used over the past three decades to study neurodegenerative disorders, particularly to recapitulate aspects of neuroinflammation (Henn et al., 2009; Timmerman et al., 2018). Their responsiveness to stimulation with lipopolysaccharides (LPS) and upregulation of key genes similar to primary microglia validated their use as a suitable substitute for primary microglia as an *in vitro* model. The NP concentration for cell treatment was selected based on the results of a MTT assay, where NPs were used at 200× dilution in culture medium to ensure at least 90% cell viability (Supplementary Figure S3). For NP uptake pathway studies, pretreatment with chlorpromazine hydrochloride, a clathrin-mediated endocytosis inhibitor, reduced NP uptake by 53%, while caveolae-mediated endocytosis and macropinocytosis inhibitors only reduce the uptake by 19 and 30% respectively (Figure 3). This suggests that the [FAA:TA] NP is primarily transported into microglia via clathrin-coated endocytic vesicles assembled on the plasma membrane, in agreement with previous publications on polymeric NPs (Papa et al., 2014). Macropinocytosis is also a dominant pathway for NP uptake, while disruption of lipid rafts via depletion of cholesterol with methyl-β-cyclodextrin did not affect uptake.

**FIGURE 4:**
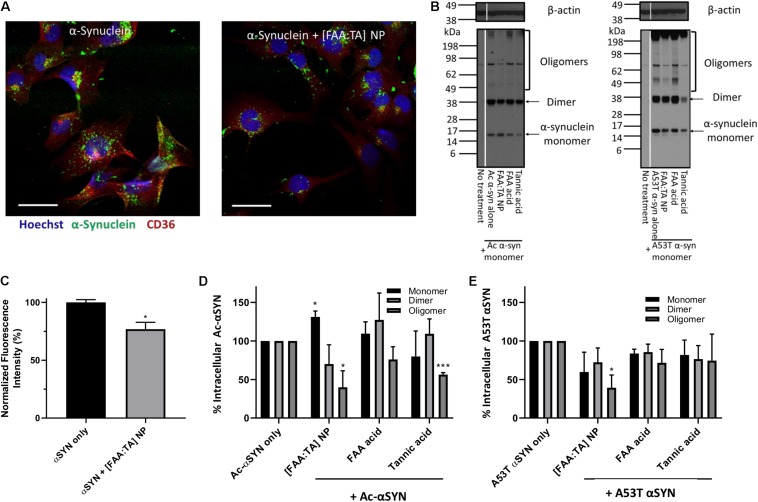
NPs’ effect on inhibiting intracellular oligomerization of N-terminal acetylated and A53T mutant αSYN **(A)** Overall intracellular αSYN uptake with and without NP treatment was evaluated with Alexa488 labeled αSYN. Microglia were visualized with nuclei stain Hoechst 33342 (Blue) and microglial scavenger receptor CD36 (Red). Scale bar = 20 μm **(B)** BV2 microglia treated with monomeric αSYN (Ac- and A53T) and NPs, were lysed after 24 h incubation to detect intracellular αSYN content. Western blot was conducted with cell lysates, staining for αSYN and beta-actin. Oligomeric αSYN was detected inside monomeric αSYN-treated microglia. A significant reduction in oligomer content was observed in NP-treated microglia, indicating that [FAA:TA] NPs can lower intracellular αSYN aggregation. **(C)** Quantified αSYN fluorescence intensity representing overall αSYN uptake in microglia. Error bar shows standard error of *n* = 3; *P = 0.0216 by one-way ANOVA. **(D,E)** αSYN monomer, dimer and oligomer bands intensity was quantified and data shown in bar graph. In **(D)**, for monomers: *P = 0.0156 for Ac-αSYN only vs. [FAA:TA] NP; for oligomers: *P = 0.04802 for Ac-αSYN only vs. [FAA:TA] NP and ***P = 0.0006 for Ac-αSYN only vs. Tannic acid by one-way ANOVA. In **(E)**, for oligomers: *P = 0.0210 for A53T αSYN only vs. [FAA:TA] NP by one-way ANOVA.

Next, we explored whether NPs can modulate αSYN content within microglia. We treated microglia with fluorescently labeled αSYN and performed immunocytochemical staining to quantify the intracellular αSYN content (Figure 4A). Quantification of fluorescence intensity in the microscopy images revealed a decrease in the overall αSYN content (Figures 4A,C). Western blot analysis was performed on microglial cell lysates to determine the effects of NP treatment on intracellular αSYN aggregate content. Here, mutant A53T αSYN was also used in addition to Ac-αSYN to induce intracellular αSYN oligomer formation in microglia, since A53T mutant αSYN is one of the most common mutant forms in found in familial PD and is considered to promote aggregation (Conway et al., 2000; Stefanis, 2012). When microglia were treated with high concentrations of these extracellular αSYN monomers, αSYN oligomers formed intracellularly (Figure 4B). For Ac-αSYN, treatment with [FAA:TA] NPs significantly decreased the presence of intracellular Ac-αSYN aggregates, particularly the oligomer content by 60% (*p* < 0.05) (Figures 4B,D). Unexpectedly, NP treatment also increased the population of intracellular monomeric Ac-αSYN by 30% (*p* < 0.05), which could be the result of the NPs’ aggregation-inhibiting action that leaves more Ac-αSYN in its non-toxic monomeric form after internalization within microglia (Figures 4B,D). No difference in dimer population was observed with NP treatment. Tannic acid treatment also significantly decreased intracellular Ac-αSYN oligomer formation (Figures 4B,D). This aligns with our cell-free fibrillization results, validating our hypothesis that tannic acid has a strong inhibitory effect on αSYN aggregation, and the inihibitory effect of NPs is likely due to the release of FAA acid and tannic acid (Supplementary Figure S4).

For mutant A53T αSYN, treatment with [FAA:TA] NPs also decreased intracellular oligomer formation to a similar extent as with Ac-αSYN by 60% (*p* < 0.05) (Figures 4B,E). However, we did not observe an increase in intracellular monomeric αSYN, which could be explained by the faster protein aggregation kinetics of A53T mutant αSYN compared to Ac-αSYN (Conway et al., 2000).

### Antioxidant NPs Attenuate Microglial Activation

**FIGURE 5:**
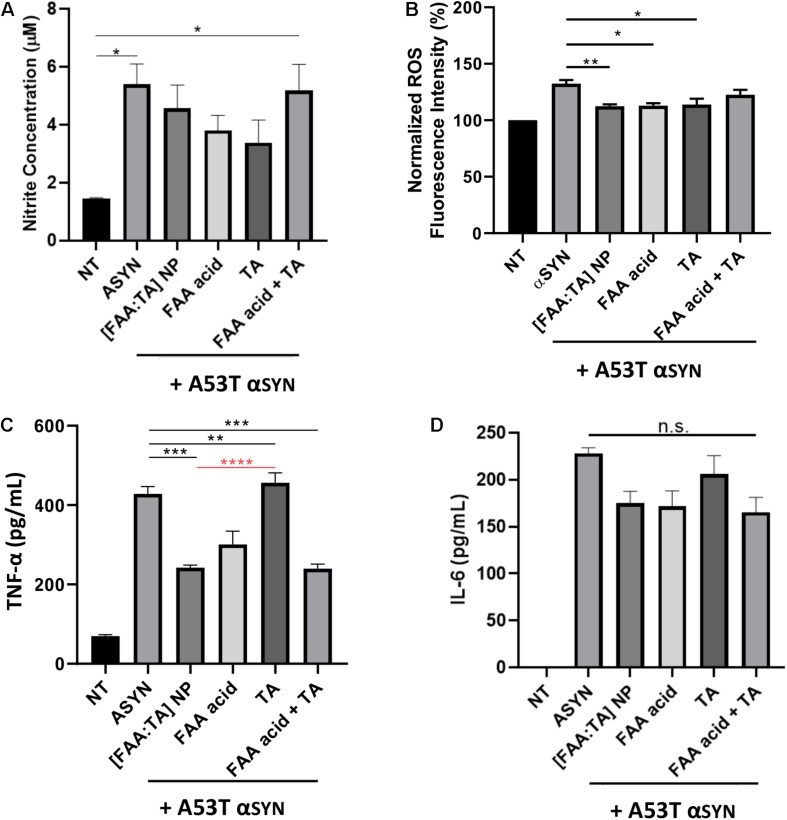
Effect of [FAA:TA] NPs on reducing microglia activation. **(A–C)** BV2 microglia was treated with 5 μM (for Griess and TNF-α) or 10 μM (for ROS) A53T αSYN in the presence of absence of NP/compounds controls. Supernatants were harvested 24 h later and concentration of Nitrite and TNF-α were measured by Griess Reagent and ELISA. Intracellular ROS was detected using CellRox Dye. **(D)** LPS primed BV2 microglia were stimulated for 24 h with 5 μM A53T αSYN with or without NP/compounds. IL-6 concentration was determined by ELISA. Data are represented as means ± SEM; *n* = 3. In **(A)**, *P = 0.0154 for NT vs. αSYN only and *P = 0.0223 for NT vs. FAA acid + TA by one-way ANOVA. In **(B)**, **P = 0.0083 for αSYN only vs. [FAA:TA] NP, *P = 0.0103 for αSYN only vs. FAA acid and *P = 0.0145 for αSYN only vs. TA by one-way ANOVA. In **(C)**, *P* values are relative to αSYN (black) and [FAA:TA] NP condition (red). ***P = 0.0002 for αSYN only vs. [FAA:TA] NP, **P = 0.0059 for αSYN only vs. FAA acid, ***P = 0.0002 for αSYN only vs. FAA acid + TA and ****P < 0.0001 for [FAA:TA] NP vs. TA by one-way ANOVA. [FAA:TA] NPs was found to significantly reduce pro-inflammatory cytokine TNF-α and intracellular ROS production.

Chronic activation of microglia results in the release of neurotoxins and inflammatory agents, which eventually results in further neuronal damage or death. Of the various neurotoxic factors released from activated microglia, free radicals as well as pro-inflammatory cytokines are particularly harmful to DA neurons, which are extremely vulnerable to oxidative damage. Previous research has shown that ferulic acid has strong anti-inflammatory properties (Srinivasan et al., 2007; Huang et al., 2011; Liu et al., 2017). Therefore, we investigated the effect of [FAA:TA] NPs on microglial NO, ROS, TNF-α, and IL-6 production upon A53T αSYN stimulation. As a stimulant, 5 μM or 10 μM A53T αSYN alone was sufficient to induce microglial activation indicated by the significant elevation of NO, ROS and TNF-α relative to the no treatment control (Figures 5A–C). For IL-6 detection in cell culture supernatant, LPS was used to prime microglia before αSYN treatment since αSYN alone failed to elevate IL-6 levels above that of the untreated control (Figure 5D).

NO production was not significantly modulated with NP treatment (Figure 5A). Interestingly, the free individual components FAA acid and tannic acid lowered nitrite concentration modestly but the control condition of a mixture of FAA acid and tannic acid molecule caused no significant change in NO production (Figure 5A). Similarly, intracellular ROS was significantly decreased with NP treatment as well as with the introduction of FAA acid and tannic acid, but the mixture of FAA acid and tannic acid did not markedly alter ROS (Figure 5B).

[FAA:TA] NP treatment significantly decreased levels of TNF-α released from microglia, indicating the NP’s ability to regulate αSYN-induced microglial activation (Figure 5C). While both FAA acid compound and the mixture of FAA acid and tannic acid were able to reduce TNF-α production significantly, the effect of tannic acid alone was significantly weaker than that of [FAA:TA] NPs (Figure 5C). The use of the NP formulation, FAA acid alone, and mixture of FAA acid and tannic acid had a modest but non-significant effect on the attenuation of IL-6 production (Figure 5D). Our results suggest that the FAA acid component plays a major and differential role in controlling pro-inflammatory cytokine production and that the NP formulation supports the anti-inflammatory action of FAA acid.

**FIGURE 6:**
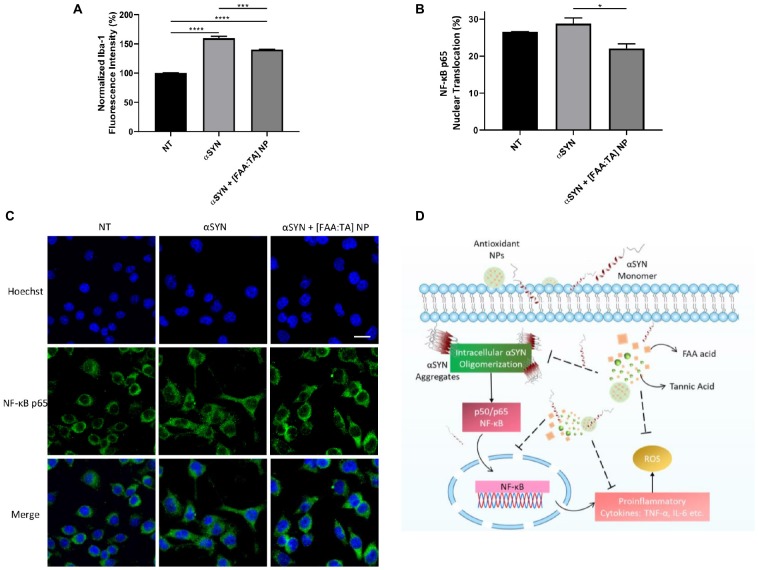
[FAA:TA] NPs reduce microglial Iba-1 expression and NF-κB nuclear translocation. **(A,B)** BV2 microglia were treated with 10 μM A53T αSYN in the presence of absence of NP/compounds controls. Cells were fixed and stained with corresponding antibodies. Data are represented as means ± SEM; *n* = 3. In **(A)** *P = 0.0147 for αSYN only vs. αSYN + [FAA:TA] NP by one-way ANOVA. In **(B)**, ****P < 0.0001 for NT vs. αSYN only and NT vs. αSYN + [FAA:TA] NP, ***P = 0.0009 for αSYN only vs. αSYN + [FAA:TA] NP by one-way ANOVA. **(C)** Representative confocal microscopy images showing NF-κB nuclear translocation. Scale bar = 20 μm. **(D)** [FAA:TA] NPs modulate microglial activation through NF-κB pathway. Intrinsically disordered αSYN protein aggregates upon association with membrane bilayer or certain membrane receptors. Aggregated αSYN has been associated with NF-κB pathway activation, which further leads to the production of pro-inflammatory cytokines and free radicals. By inhibiting intracellular αSYN aggregate formation, NPs may suppress translocation of the key activation mediator (NF-κB p65) involved in αSYN-induced microglial activation. This suppression leads to reduction in downstream pro-inflammatory cytokines TNF-α and IL-6 as well as neurotoxic free radical ROS and NO production.

The effect of [FAA:TA] NPs on the modulation of microglial activation was further confirmed by quantifying the Iba1 fluorescence intensity of immunostained BV2 microglia. NP treatment of αSYN-stimulated microglia was able to reduce the expression of Iba1, which indicates a decrease in the extent of microglial activation (Figure 6A). To further investigate the mechanism by which the NPs influence αSYN-induced microglial activation, a NF-κB nuclear translocation assay was conducted on stimulated microglia. The nuclear translocation of NF-κB has been shown to promote the expression of TNF-α and the release other pro-inflammatory cytokines in microglia (Ferreira and Romero-Ramos, 2018; Shao et al., 2019). αSYN treatment slightly increased the NF-κB nuclear translocation above basal level although not significantly (Figures 6B–C). BV2 microglia have been shown to have elevated baseline levels of activation, which may indicate why no difference was seen here. [FAA:TA] NP treatment significantly reduced the nuclear translocation of NF-κB in αSYN-treated microglia (Figures 6B,C), which has the effect of inhibiting the αSYN-induced inflammatory signaling response, leading to attenuation of the production of pro-inflammatory cytokines and ROS as described above.

## Discussion

Currently, there is no existing effective therapy to reverse the progression of PD, although a number of therapeutic approaches are used in clinical practice to improve motor symptoms (Lang and Espay, 2018). The improvements in motor symptoms with drugs such as L-DOPA or non-pharmacological approaches such as deep brain stimulation, unfortunately, are transient in nature since αSYN aggregation and accumulation, and microglial activation involved in the neuroinflammation phenomena persist throughout PD progression (Rascol et al., 2002). In this work, we hypothesize that modulating microglial-mediated intracellular αSYN oligomerization and microglial activation are key targets to design therapeutics for PD and other neurodegenerative diseases.

We demonstrated that both FAA acid and tannic acid components of our antioxidant NP formulation inhibit αSYN fibrillization. Ferulic acid-derived diacid molecule was used because previous studies have shown the strong anti-inflammatory properties of ferulic acid in microglial cell systems and in neuroinflammatory mouse models, where ferulic acid inhibited glial cell activation and reversed neuronal synaptic dysfunction (Huang et al., 2011; Takahashi et al., 2015; Liu et al., 2017; Rehman et al., 2018). Tannic acid molecule was selected due to its strong inhibitory effect on αSYN multimeric oligomer formation and prevention of neurotoxic amyloid fibril formation (Caruana et al., 2011; Braidy et al., 2017). Our observations are in line with previous work on antioxidant polyphenols’ anti-fibrillogenic ability including tannic acid, nordihydroguaiaretic acid (NDGA) and gallic acid (Ono and Yamada, 2006; Caruana et al., 2011). Ferulic acid is also one of the anti-fibrillogenic antioxidants that has previously been identified but is reported to be less potent than tannic acid, similar to our results (Supplementary Figure S2; Ono and Yamada, 2006). We found that FAA acid has much higher potency in inhibiting fibrillization compared with ferulic acid for both wild type and N-terminal acetylated αSYN. By incorporating adipic acid linker within two ferulic acid molecules, the hydrophilicity is slightly decreased (Ouimet et al., 2013). Considering the fact that hydrophobic interactions between phenolic compounds and randomly coiled protein likely initiates a phenol-protein complex formation, increased hydrophobicity could enable a strong hydrophobic interaction between FAA acid molecule and the hydrophobic domain of αSYN protein. The middle hydrophobic region of αSYN is critical for αSYN to polymerize into amyloid fibrils and is a major contributor to the formation of pathological inclusions (Waxman et al., 2009). Tannic acid contains 5 gallol groups and 5 catechol groups and these hydroxyl-rich moieties greatly contribute to tannic acid-αSYN association via the formation of multiple hydrogen bonds. In addition, the π-π stacking between aromatic rings on TA and aromatic side chains on αSYN has also been associated with TA’s inhibition on αSYN fibrillization (Stefani and Rigacci, 2013; Ardah et al., 2014). There was no difference observed in the anti-fibrillogenic effect of NPs on wild type αSYN and N-terminal acetylated αSYN (Figure 2 and Supplementary Figure S1). N-terminal acetylated αSYN is the most prevalent form of αSYN in mammals and has stronger membrane interactions due to stabilized N-terminal helicity (Kang et al., 2012). Future studies could be done to elucidate the molecular binding mechanism between tannic acid, FAA acid and αSYN with different post-translational modifications and determine how the NP structural properties may affect individual molecules’ association with αSYN.

While the majority of PD cases are idiopathic, 5% of PD are familial and are linked to point mutations in the SNCA gene. Among missense mutations in SNCA, A53T appears to be the most prevalent one and has shown faster aggregation kinetics toward oligomerization than wild type αSYN (Conway et al., 2000). We chose to use A53T mutant αSYN to induce microglial-mediated intracellular oligomerization and challenge the NPs’ aggregation-inhibiting ability with a more aggressive aggregation profile. The significant reduction in the intracellular αSYN oligomer population with NP treatment in microglia challenged with Ac αSYN and A53T αSYN suggests that the binding of tannic acid to αSYN has faster kinetics than αSYN-αSYN self-aggregation, which matches with our observation in the cell-free fibrillization assay (Figures 4D,E). There is a decrease in the overall αSYN uptake in microglia with the addition of NPs, which suggests that the NPs may be interfering with certain receptor-mediated interactions between αSYN and microglia. Alternatively, there is also the possibility that the FAA acid component of the NPs could be interacting with αSYN in the extracellular space and thus slowing down its internalization.

While many small molecules have been investigated for their use in modulating microglial activation through different molecular pathways, without properly engineered delivery mechanisms, these drugs may lack stability to ensure their continued bioactivity and may also disrupt the normal function of other cells sharing the same biomarkers (Choi et al., 2011; Song and Suk, 2017). In this study, we investigated the role of a NP formulation to potentially enable improved stability of antioxidant components and sustained release of active compounds. We hypothesized that the combination of FAA acid and tannic acid could have potentially complementary effects on microglial activation and its inflammatory phenotype. Tannic acid, as an example, is a strong antioxidant and has been widely recognized for its anti-inflammatory, anti-carcinogenic and anti-bacterial effects (Braidy et al., 2017). Despite its promise, tannic acid’s efficacy via direct administration is limited due to the high potential of binding with proteins and formation of large complexes via chelation with metal ions (Purawatt et al., 2007; Braidy et al., 2017). While the incorporation of tannic acid onto protein and peptide-based therapeutics and polymeric nanoparticles has shown better cellular and tissue-level targeting capabilities, the delivery of therapeutics to the CNS penetrating through the blood-brain barrier (BBB) remains challenging even for nanomaterials (Abouelmagd et al., 2016; Shin et al., 2018; Saeedi et al., 2019). In order for the [FAA:TA] NPs to achieve *in vivo* efficacy in inhibiting αSYN oligomerization and reducing pro-inflammatory cytokine production, the NP structure can be modified via polyethylene glycol (PEG) coating to enable better circulation, and via bioconjugation to alter surface charge or add targeting ligands to enhance its performance *in vivo* and to provide targeted delivery to microglia in the CNS (Saeedi et al., 2019).

The design of using composite NPs composed of ferulic acid derived diacid molecule and tannic acid was able to effectively modulate the production of pro-inflammatory cytokines TNF-α and IL-6, NO and ROS production from αSYN-stimulated microglia, which has been shown to cause neurotoxicity (Marin-Teva et al., 2011). Previous research has shown ferulic acid’s efficacy in ameliorating microglial activation induced by β-amyloid peptide in a mouse model and in LPS-challenged BV2 microglia (Kim et al., 2004; Huang et al., 2011). However, the efficacy of ferulic acid may be limited by its lack of stability in different pHs, temperature conditions, and dosage requirements (Wang et al., 2011). To improve the physiochemical stability, ferulic acid was conjugated via an adipic linker which enables improved stability and sustained release of ferulic acid, protecting the carboxylic acid from decarboxylation (Ouimet et al., 2013, 2015). The lack of a detectable effect of free FAA acid and tannic acid mixture control in controlling nitrite release might suggest that the presence of tannic acid may interact with FAA acid in solution before either binds to αSYN (Figure 5A). This could lower the availability of FAA acid in solution and also prevent FAA acid from scavenging free radicals in the extracellular region. The reason for the reduction in the release of pro-inflammatory cytokines with NP treatment may be twofold. First, the NPs directly associate with extracellular αSYN to prevent toxic oligomer formation; second, FAA acid and tannic acid components within internalized NPs suppress the expression of key signaling-pathway molecule(s) involved in intracellular microglial activation.

Although tannic acid treatment reduced nitrite production by 38% from αSYN-challenged microglia, this was not a statistically significant decrease, which could be due to the nitrite concentration approaching the detection limit of the Griess Reagent (2.5 μM) (Figure 5A). While previous research has shown that tannic acid can reduce the production of neuroinflammatory factors such as ROS, NO and TNF-α from LPS-treated microglia via the suppression of the NF-κB pathway, our results show that tannic acid only reduces intracellular ROS production but does not have an appreciable effect on the other two markers (Figures 5A–D; Wu et al., 2019). This could potentially suggest that the rate at which tannic acid associates with αSYN is much faster than its interaction with other signaling molecules involved in the pathway (Shaham-Niv et al., 2018). The only major inflammatory biomarker that was not appreciably modulated by the [FAA:TA] NPs was IL-6 production from LPS-primed microglia (Figure 5D). This result was likely related to the inability of αSYN alone to elevate IL-6 levels in the absence LPS. The lack of action of [FAA:TA] NPs in modulating αSYN-induced activation of LPS-primed microglia may indicate that the effect of NPs on modulating microglial activation is more heavily dependent on its action on preventing the formation of intracellular αSYN aggregates rather than its effect on direct suppression of intermediate signaling markers involved.

The NF-κB pathway has been shown to be involved in the activation of microglia upon αSYN treatment, which leads to the formation of free radicals and pro-inflammatory cytokines (Kim et al., 2013; Ferreira and Romero-Ramos, 2018; Shao et al., 2019). Our results showed that [FAA:TA] NPs can significantly reduce the nuclear translocation of NF-κB induced by αSYN stimulation (Figures 6B,C). This indicates that the NPs’ action in modulating the release of pro-inflammatory cytokines and ROS may primarily be through the NF-κB pathway, which is activated by the intracellular oligomerization of αSYN upon association with microglia. Since the production of ROS and pro-inflammatory cytokines may in turn also provoke the translocation of NF-κB to the nucleus, the [FAA:TA] NP could potentially have intercepted this cycle of microglial activation (Figure 6D). The mechanism by which [FAA:TA] NPs modulate neuroinflammation could potentially inform the design of alternative therapeutics with better targeting capabilities and efficacy for treatment of PD.

As previously discussed, rather than focusing on neurons as a pharmacological target, microglia could serve as a more upstream cellular target of interest in PD since microglia are involved in αSYN trafficking, aggregation, and clearance, and are closely associated with the triggers for the neuroinflammation process (Ferreira and Romero-Ramos, 2018; Moore et al., 2018; Xia et al., 2019). Further investigation into how antioxidant-based composite NPs may influence αSYN transmission among microglia and other CNS cells, especially neurons, would provide valuable insight into the interplay between different CNS cell types in the trafficking and aggregation of αSYN and its related pathology. While BV2 microglia are used here as an *in vitro* model for αSYN-induced activation, the use of antioxidant-based NP formulations cannot be established without thorough investigation within PD animal models (Visanji et al., 2016; Thakur et al., 2017). Future studies will need to be conducted in PD murine models with αSYN over-expression, investigating the possible route of NP administration, the stability of NPs *in vivo*, and whether sustained release of the active therapeutic components ferulic acid and tannic acid can be achieved.

## Conclusion

In summary, antioxidant NPs composed of ferulic acid diacid molecule and tannic acid can be used to concertedly modulate two critical pathological aspects of PD: (1) the intracellular αSYN aggregation in microglia, which leads to neurotoxicity and (2) the oxidative stress caused by microglia during neuroinflammation in PD. This work provides a promising antioxidant-based nanotherapeutic to target pathological protein aggregation and neuroinflammation in neurodegenerative diseases.

## Data Availability Statement

The datasets generated for this study are available on request to the corresponding author.

## Author Contributions

NZ designed and performed the experiments, analyzed the data, prepared the figures, and wrote the manuscript. XY, HC, and YC performed the experiments. NF designed and performed the experiments, contributed to the data analysis, and edited the manuscript. RC designed and performed experiments. LJ designed experiments. KU and PM contributed to data analysis, edited the manuscript, and provided financial support and supervision. ZP and JB provided financial support and supervision.

## Conflict of Interest

The authors declare that the research was conducted in the absence of any commercial or financial relationships that could be construed as a potential conflict of interest.
