# Supplementary figures and images for: Antioxidant Nanoparticles for Concerted Inhibition of α-Synuclein Fibrillization, and Attenuation of Microglial Intracellular Aggregation and Activation
Source: Front Bioeng Biotechnol. 2020 Feb 21;8:112. doi: 10.3389/fbioe.2020.00112 (PMC7046761; doi:10.3389/fbioe.2020.00112)

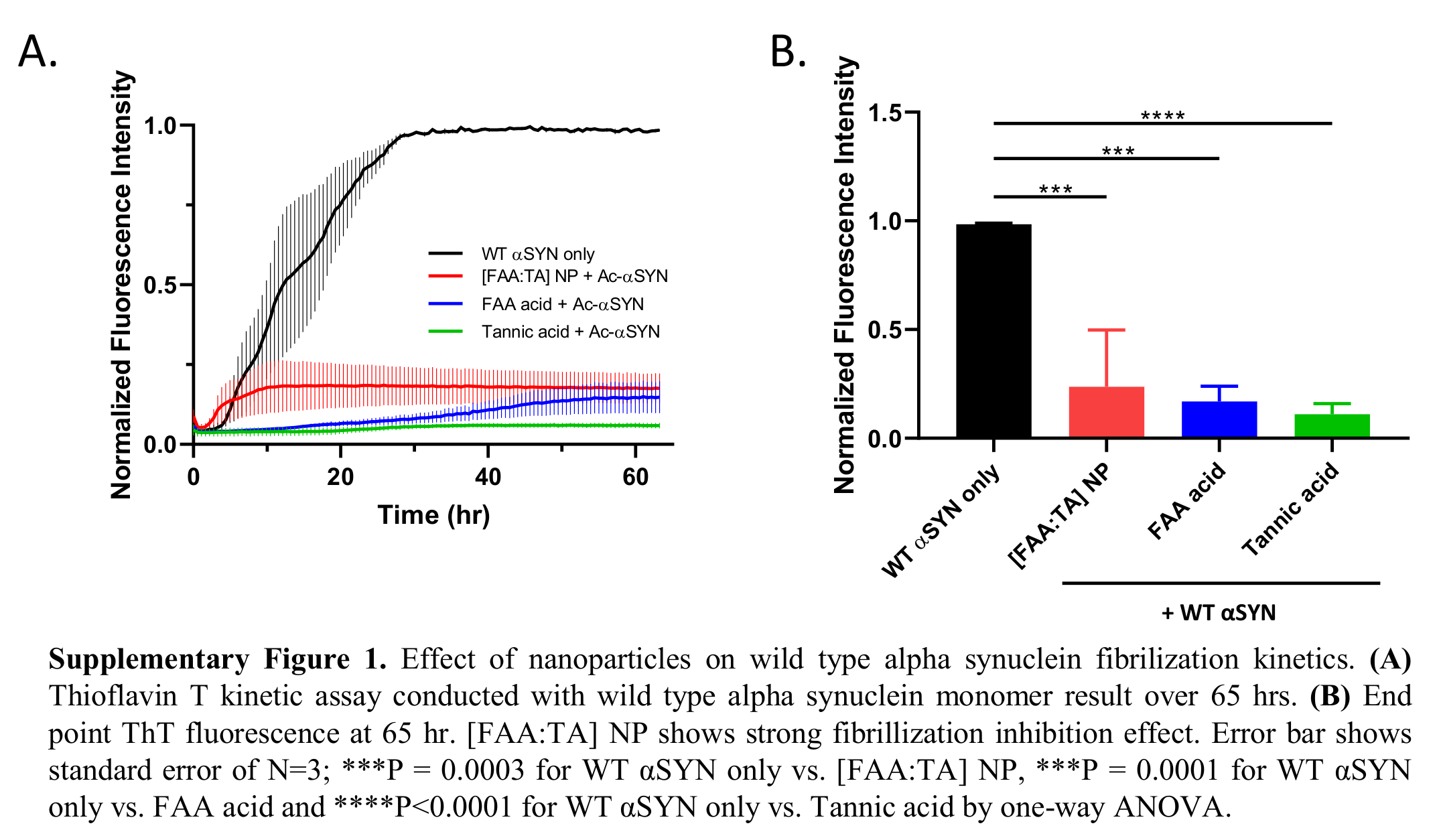

Supplement: Supplementary file 1 [file Image_1.TIF]

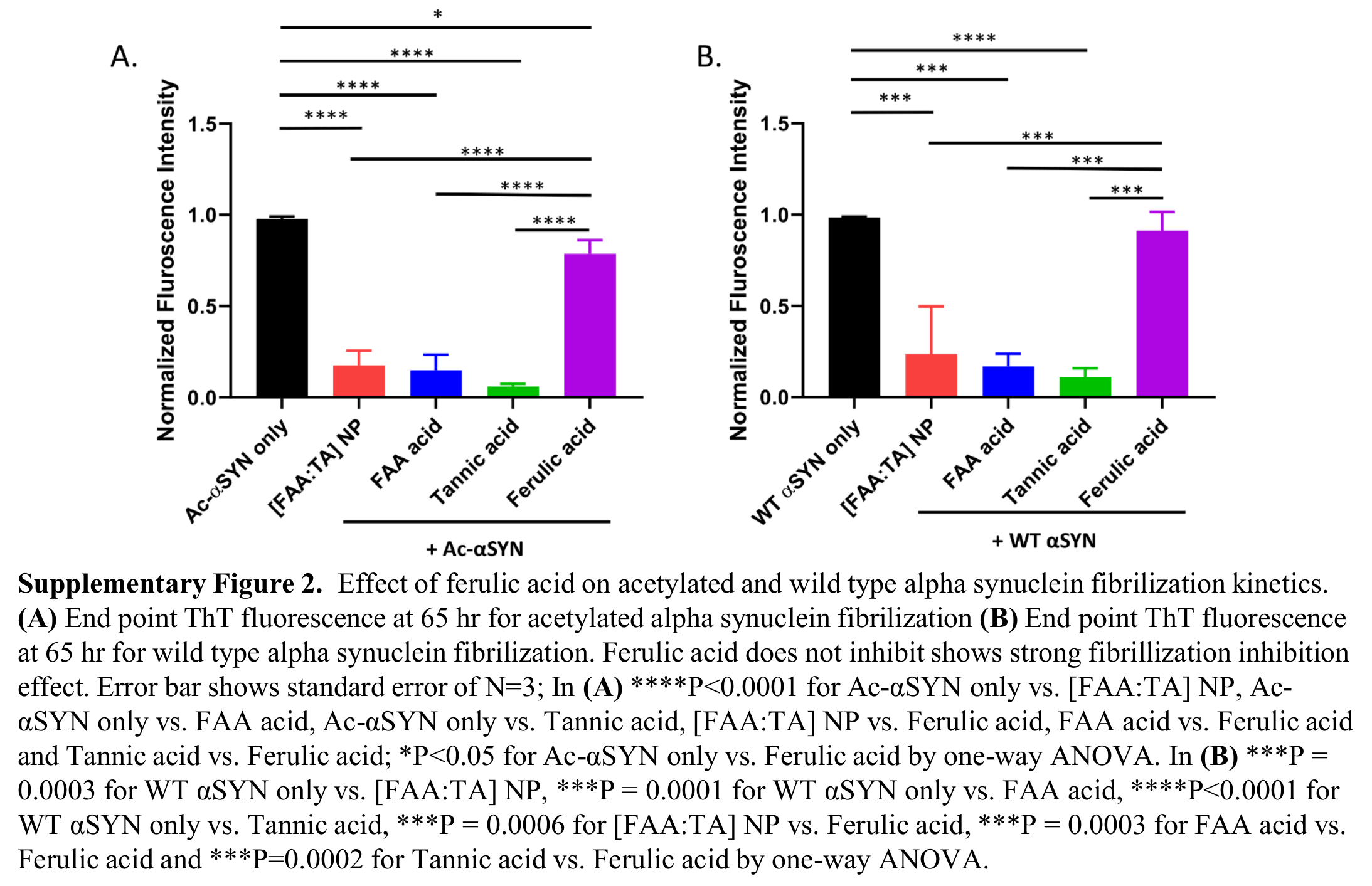

Supplement: Supplementary file 2 [file Image_2.TIF]

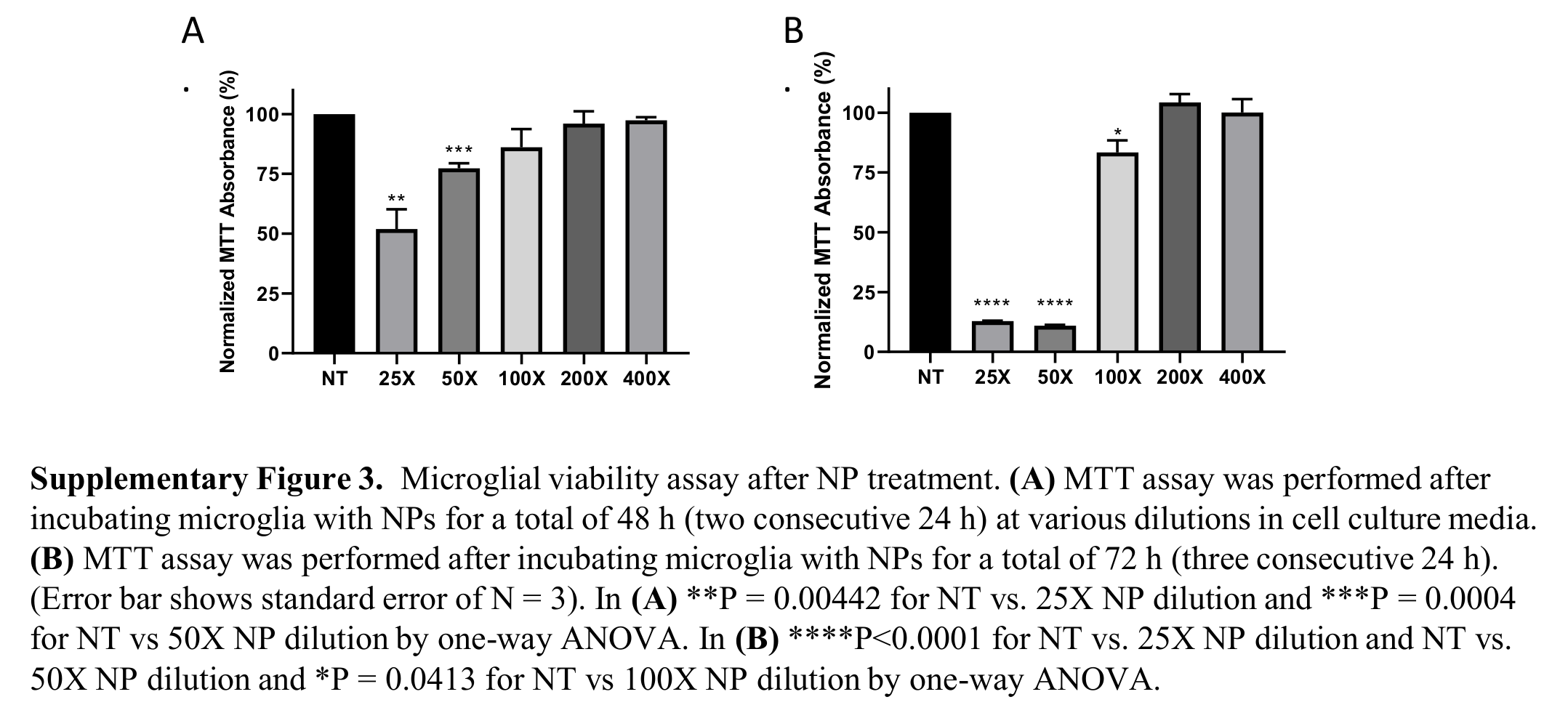

Supplement: Supplementary file 3 [file Image_3.TIF]

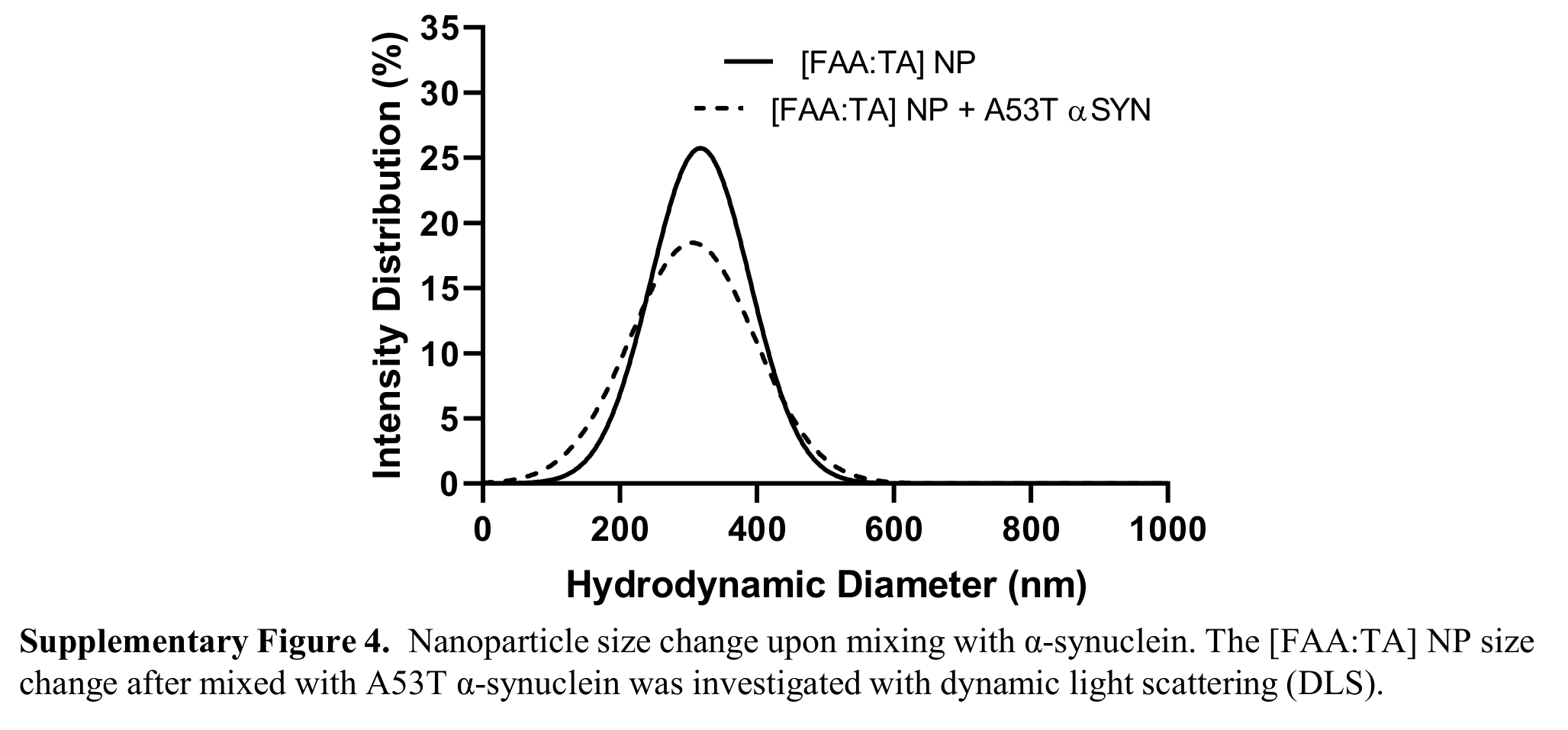

Supplement: Supplementary file 4 [file Image_4.TIF]
